# Contribution of Rare and Low-Frequency Variants to Multiple Sclerosis Susceptibility in the Italian Continental Population

**DOI:** 10.3389/fgene.2021.800262

**Published:** 2022-01-03

**Authors:** Ferdinando Clarelli, Nadia Barizzone, Eleonora Mangano, Miriam Zuccalà, Chiara Basagni, Santosh Anand, Melissa Sorosina, Elisabetta Mascia, Silvia Santoro, PROGEMUS, P Crociani, PROGRESSO, F Esposito, Franca Rosa Guerini, Eleonora Virgilio, Antonio Gallo, Alessandro Pizzino, Cristoforo Comi, Vittorio Martinelli, Giancarlo Comi, Gianluca De Bellis, Maurizio Leone, Massimo Filippi, Federica Esposito, Roberta Bordoni, Filippo Martinelli Boneschi, Sandra D'Alfonso

**Affiliations:** ^1^ Laboratory of Human Genetics of Neurological Disorders, Division of Neuroscience, IRCCS San Raffaele Scientific Institute, Milan, Italy; ^2^ Department of Health Sciences, UPO, University of Eastern Piedmont, and CAAD (Center for Translational Research on Autoimmune and Allergic Disease), Novara, Italy; ^3^ Institute for Biomedical Technologies, National Research Council of Italy, Segrate, Italy; ^4^ Department of Informatics, Systems and Communications (DISCo), University of Milano-Bicocca, Milan, Italy; ^5^ IRCCS Fondazione Don Carlo Gnocchi, Onlus, Milan, Italy; ^6^ Department of Translational Medicine, Section of Neurology and IRCAD, UNIUPO, Novara, Italy; ^7^ MS Center, I Division of Neurology, Department of Advanced Medical and Surgical Sciences (DAMSS), University of Campania “Luigi Vanvitelli”, Naples, Italy; ^8^ Neurology Unit and Neurorehabilitation Unit, IRCCS San Raffaele Scientific Institute, Milan, Italy; ^9^ Vita-Salute San Raffaele University, Milan, Italy; ^10^ Neurology Unit, Fondazione IRCCS Casa Sollievo Della Sofferenza, San Giovanni Rotondo, Italy; ^11^ Neuroimaging Research Unit, Division of Neuroscience, IRCCS San Raffaele Scientific Institute, Milan, Italy; ^12^ Neurophysiology Service, IRCCS San Raffaele Scientific Institute, Milan, Italy; ^13^ Department of Pathophysiology and Transplantation (DEPT), Dino Ferrari Centre, Neuroscience Section, University of Milan, Milan, Italy; ^14^ Neurology Unit, MS Centre, Foundation IRCCS Ca’ Granda Ospedale Maggiore Policlinico, Milan, Italy

**Keywords:** multiple sclerosis, rare variants, EFCAB13, pool sequencing, burden test

## Abstract

Genome-wide association studies identified over 200 risk loci for multiple sclerosis (MS) focusing on common variants, which account for about 50% of disease heritability. The goal of this study was to investigate whether low-frequency and rare functional variants, located in MS-established associated loci, may contribute to disease risk in a relatively homogeneous population, testing their cumulative effect (burden) with gene-wise tests. We sequenced 98 genes in 588 Italian patients with MS and 408 matched healthy controls (HCs). Variants were selected using different filtering criteria based on allelic frequency and *in silico* functional impacts. Genes showing a significant burden (n = 17) were sequenced in an independent cohort of 504 MS and 504 HC. The highest signal in both cohorts was observed for the disruptive variants (stop-gain, stop-loss, or splicing variants) located in *EFCAB13*, a gene coding for a protein of an unknown function (*p* < 10^–4^). Among these variants, the minor allele of a stop-gain variant showed a significantly higher frequency in MS versus HC in both sequenced cohorts (*p* = 0.0093 and *p* = 0.025), confirmed by a meta-analysis on a third independent cohort of 1298 MS and 1430 HC (*p* = 0.001) assayed with an SNP array. Real-time PCR on 14 heterozygous individuals for this variant did not evidence the presence of the stop-gain allele, suggesting a transcript degradation by non-sense mediated decay, supported by the evidence that the carriers of the stop-gain variant had a lower expression of this gene (*p* = 0.0184). In conclusion, we identified a novel low-frequency functional variant associated with MS susceptibility, suggesting the possible role of rare/low-frequency variants in MS as reported for other complex diseases.

## Introduction

Multiple sclerosis (MS; MIM 126200) is a disease of the central nervous system (CNS) characterized by chronic inflammation, demyelination, and axonal loss (Hauser and Oksenberg, 2006). It is a complex multifactorial disorder, with both genetic and environmental components playing a role in disease susceptibility. Genome-wide association studies (GWASs) greatly helped to elucidate MS genetic risk, revealing a highly polygenic architecture for the disease, with an ever-increasing number of common SNPs associated to risk: the recent large-scale study of the International Multiple Sclerosis Genetics Consortium (IMSGC) on more than 100,000 individuals provided robust evidence for association of 200 autosomal risk loci outside the HLA region, one locus on chromosome X and 31 HLA loci in addition to the well-known HLA-DRB1*15:01 (International Multiple Sclerosis Genetics Consortium, 2019). Despite this success and similarly to other complex diseases, a major fraction of heritability for MS still remains unexplained by this set of loci, raising the well-known issue of missing heritability (Manolio et al., 2009). Hypothesized sources of hidden genetic risk include an inherently imperfect tagging in current GWAS of unobserved causal variants, structural variation, gene–gene and gene–environment interactions, epigenetic effects, and a role for rare variants with weak to moderate effects (Goris and Liston, 2012).

Rare variants, which due to purifying selection is enriched in protein impacting variants (Gorlov et al., 2008), is not well captured by widely used array-based platforms. Furthermore, its investigation is hampered by the fact that very large sample sizes are needed to achieve sufficient power. In the MS field, such a large-scale effort has been conducted on individual protein-coding variation in the context of IMSGC, which recently identified six low-frequency coding variants contributing to MS risk (International Multiple Sclerosis Genetics Consortium, 2018). On the other hand, next-generation sequencing (NGS) provides a comprehensive assay for the study of all variants.

The goal of this study was to evaluate the role in MS risk of protein-altering and putative regulatory low-frequency (Minor Allele Frequency, MAF<5%) and rare variants (MAF<1%) in a cohort of subjects of Italian continental ancestry using an NGS approach. Due to power constraints, we did not perform an unbiased genome-wide scan but rather selected genes in candidate regions, chosen among those emerging from MS GWAS studies. We selected a cost-effective technique by performing sequencing on pools of individuals by taking advantage of available data analysis tools for the identification of rare variants (Schlotterer et al., 2014).

Leveraging pool sequencing, we thus performed targeted sequencing in a discovery cohort of 588 MS patients and 408 matched healthy controls (HCs). We adopted gene-based tests to assess the cumulative effect of multiple variants within a gene, a strategy that has been increasingly applied to sequencing data to improve detection of genetic risk signals (Lin and Tang, 2011; Do et al., 2012; Lee et al., 2014). Since different genes may have a specific genetic architecture linking them to MS risk, we tested various combinations of variants and sought to replicate the findings in independent cohorts and assayed with both pool sequencing and array-based platforms. Finally, we functionally characterized the disruptive variant showing the most consistent pattern of association across three different cohorts, located in the *EFCAB13* gene, which emerged as the most associated gene in the discovery cohort.

## Materials and Methods

### Subjects

MS patients and HC were recruited by several clinical centers, from Continental Italy, mainly from northern Italy, part of three consortia (PROGEMUS, PROGRESSO, and HYPERGENE). MS patients were diagnosed according to McDonald criteria. All recruited subjects signed a written informed consent for the conduction of genetic studies in the MS field for research purposes. Patients and HC with Sardinian ancestry were excluded from the analysis.

Patients and controls derived from three cohorts:

*1*) *Discovery cohort:* 588 MS patients and 408 HC, pooled in 84 groups of 12 samples each (49 MS pools, 34 HC pools). The discovery cohort was constituted of patients showing the highest genetic burden of known common MS susceptibility variants, as summarized by a weighted genetic risk score (wGRS), calculated as previously described (De Jager et al., 2009). The healthy controls were individually matched with MS patients, according to sex and ancestry as detected by principal components (PCs), and with no selection according to wGRS values. Ancestry matching was performed considering the first two PCs, calculated on genotyping data derived from either Illumina® ExomeChip custom array or Illumina® Human 660 Quad platforms, and eigenvectors were compared by binning PC scores in categories of a 0.01 width.

*2*) *Replication cohort:* 504 MS patients and 504 HC, pooled in 84 groups of 12 individuals each (42 MS pools, 42 control pools). To extend generalizability of the results to a wider population, the criterion based on wGRS genetic burden was not applied for the selection of MS patients in this cohort.

*3*) *Array cohort*: for the genes emerging from the replication step, we investigated the contribution of their variants by constituting a completely independent cohort of 2,728 subjects (1298 MS and 1430 HC) individually genotyped on Illumina® array platforms. More precisely, the cohort was composed of 805 HC genotyped on the Illumina® ExomeChip custom array (International Multiple Sclerosis Genetics Consortium, 2018) and 403 MS patients and 625 HC genotyped on an Illumina® MSchip custom array (International Multiple Sclerosis Genetics Consortium, 2019), and quality controls for these two datasets were performed centrally at the IMSGC consortium site. The remaining 895 MS subjects were genotyped on an Illumina® Infinium OmniExpress and later imputed against a 1,000 Genomes phase 3 ALL reference panel: the post-QC set comprised markers with MAF>1% and Rsq >0.8.

### Selection of Target Regions

Our candidate region selection from MS-associated loci generated 46 target regions that spanned a total of 1.9 Mb and included 98 genes that were sequenced in the discovery phase: for 26 of these genes, we also sequenced the intronic and flanking regions, while for the remaining 72, we sequenced the coding and 3′UTR and 5′UTR regions including 10 base-pairs intron–exon boundaries (Supplementary Table S1).

### Pooled Sequencing

Genomic DNA samples were purified from peripheral whole blood using standard techniques. DNA pools were prepared with accurate balancing of the DNA amount of each individual so that each genome was equally represented. DNA concentrations were measured with a fluorometer method (Qubit 4.0, Thermo Fisher, Milan, Italy), and 400 ng of each DNA of 12 different individuals was pooled together for each DNA pool. For each DNA pool, libraries were prepared for both discovery and replication cohorts with the SureSelectXT Target Enrichment System for an Illumina® Paired-End Multiplexed Sequencing Library (Agilent Technologies®, Santa Clara, CA), according to the manufacturer’s protocol.

The discovery cohort was sequenced performing a paired-end (PE) multiplexed sequencing on an GaIIx platform (Illumina®, San Diego, CA), combining six pools tagged with different index sequences in each lane and producing 2 × 85 bp read lengths.

Samples from the replication cohort were sequenced performing paired-end multiplexed sequencing on an Illumina® NextSeq 500 (Illumina®, San Diego CA) platform as part of a larger panel, producing a 2 × 150 bp read length.

### Bioinformatic Pipeline

The same bioinformatic pipeline was employed both for the discovery and replication pool-sequenced cohorts.

#### Quality Control and Alignment

The FastQC tool (Andrews, 2015) was used to check the raw reads for quality, and the QC-checked paired-end reads of each pool were mapped to the human reference genome (NCBI build GRCh37) using Burrows- Wheeler Aligner BWA v0.7.5 (Li and Durbin, 2009), allowing for a maximum of three mismatches. We used *samtools* (Li et al., 2009) to remove the duplicate reads generated by PCR amplification during library preparation. Unmapped reads and reads with mapping quality (MAPQ) lower than 15 were removed. Pools showing a low depth or low coverage of target regions were resequenced: one of the MS pools of the discovery experiment did not reach a minimum depth and was removed from the subsequent analysis, leading to a set of 588 MS patients analyzed in the discovery cohort.

#### Variant Calling

Variants were called using the CRISP (v27122013) caller (Bansal, 2010), a tool specifically designed for pooled samples. For each variant in each pool, we extracted the maximum likelihood estimate for the allele counts for the alternative allele (MLAC) and used this estimate for downstream analyses. To remove spurious ALT allele reads possibly coming from sequencing errors, which can confound detection of alleles in the low-frequency spectrum, we applied appropriate filters with an empirically determined threshold to single-pool ALT allele frequencies. The thresholds were determined as described in Anand et al., 2016 and set to 2.6% and 2.4% for discovery and replication cohorts, respectively.

#### Annotation

For genomic and functional annotation, we used ANNOVAR (v. 2015Dec14) (Wang et al., 2010). Variants were annotated for gene-based annotation (refGene table), prediction of the putative effect of the missense variants (table ljb26_all), and ENCODE annotations were used to identify variants located in regions with a possible regulatory role.

The MS patients of the discovery cohort (N = 588) had been previously individually genotyped either with the Illumina® Human 660-Quad chip or with the Illumina® Immunochip platform. To validate the accuracy of our pipeline, we compared AF for variants covered in both pool-seq and genotyping platforms with AF as estimated in the pools and observed a high correlation (*R* ^2^ = 0.987). Similarly, we also observed a high correlation between pooled AF and frequencies reported in public databases (1,000 genomes_EUR *R* ^2^ = 0.980, ExAC *R* ^2^ = 0.970). We estimated allele frequency on both patients and controls, as previously described (Anand et al., 2016).

Furthermore, we performed a validation with Sanger sequencing of 12 variants, including 10 rare variants, observed in only one pool and not reported in public databases and two low-frequency variants, each reported in two pools. For each variant, we performed individual Sanger sequencing on all the 12 samples of each pool for a total of 14 pools. For all the 12 variants, Sanger sequencing confirmed both the presence of the variant and the respective allelic frequency estimated in the pool.

We applied a strict quality control filter for high-confidence SNVs (Single Nucleotide Variants) in order to remove false positives, retaining only those with a phred-scale call quality score >100.

### Qualifying Variants

We investigated contribution to MS susceptibility of rare and low-frequency synonymous, non-synonymous, disruptive and putative regulatory SNVs in target regions. Regulatory SNVs were defined based on their physical overlap with putative regulatory elements as available in region-based annotation supplied by ANNOVAR. Furthermore, we utilized a set of algorithms to predict the potential deleteriousness of a missense variant as available in ANNOVAR table ljb26_all (Wang et al., 2010). Overall, we adopted the following seven filtering criteria:

1. Disr: SNVs classified as disruptive (stop-loss, stop-gain, splice site); since we detected only 17 disruptive SNVs, with the most common having MAF = 0.0856, as estimated in our cohort, we opted for testing the contribution of disruptive variants without imposing any frequency cutoff on this filter;
2. MisDisr_01: SNVs classified as missense or disruptive, MAF<1%;
3. MisDisr_05: SNVs classified as missense or disruptive, MAF<5%;
4. DamDisr_01: SNVs classified as damaging or disruptive, MAF<1%; we analyzed missense SNV based on its categorical putative impact predicted by six tools: SIFT (D: Deleterious, score<0.05), PolyPhen2 (D: Probably Damaging or P: Possibly Damaging), MutationTaster (D: disease causing or A: disease causing automatic), MutationAssessor (M or H: functional), LRT (D: Deleterious), and FATHMM (D: Damaging). To increase the confidence level of predictions, we rated as damaging SNVs those predicted as protein damaging by at least two out of the six tools.
5. DamDisr_05: SNVs classified as damaging or disruptive, MAF<5%;
6. SynMisDisr_01: SNVs classified as synonymous, missense, or disruptive, MAF<1%;
7. Reg_01: non-coding SNVs, MAF <1%, located in introns, 3’/5’ UTR, and 1 kb upstream/downstream with overlap with the following elements annotated by Annovar software: Transcription factor binding site (tfbsConsSites table, based on Transfac Matrix database), variants disrupting microRNAs and snoRNAs (wgRna table, based on the mirbase and snoRNA database), variants disrupting predicted microRNA binding sites (targetScanS table, based on TargetScanHuman database), variants located in enhancer regions and variants disrupting enhancers, repressors, and promoters according to ENCODE annotation from the chromatin state prediction in the GM12878 cell line (Active_Promoter, Strong_Enhancer, and Insulator).

### Gene-Based Test

The joint contribution of minor alleles within genes harboring at least two of the filtered SNVs was evaluated with the burden and variance component tests. We used the weighted sum statistics (WSS) burden test (Madsen and Browning, 2009), which is sensitive in scenarios where all variants are associated with the disease in the same direction; the variance component test was the C-alpha test (Neale et al., 2011) which, by assessing the distributions of alleles, more flexibly allows for SNV with the bidirectional effect and is less sensitive to the presence of neutral variants. We empirically computed *p*-values by means of a permutation-based procedure with 10,000 random swaps of the case-control status using the pool as a statistical unit by appropriate modification of functions *WSS* and *CALPHA* implemented in R package AssotesteR (https://cran.r-project.org/web/packages/AssotesteR). We then sought to account for different genetic architectures in genes by applying combined testing, which has been found to exhibit improved power when compared to using either gene-wise test separately (Derkach et al., 2013). We combined the contribution of both tests by means of Fisher’s test statistic (Greco et al., 2016), defined as *F = -log*(*p*_*WSS*_)*-log*(*p*_*CALPHA*_)*.* Since the two tests are not independent, F statistic does not follow an asymptotic χ^2^ and its *s*ignificance was assessed with a permutation scheme as described in Greco et al., 2016 to control for correlation between the two tests and generate an appropriate null distribution. The combined test was used for the selection of genes in the discovery cohort to follow up in the replication cohort: we took an exploratory approach by following in replication genes associated at the nominal level (*p* < 0.05).

The same two tests implemented in AssotesteR were also used for the follow up of *EFCAB13* gene-disruptive variants on array-based data.

For one of the disruptive variants located in *EFCAB13* showing consistent association across the two pool-seq cohort and the array-based cohort, an inverse-variance weighting meta-analysis was conducted under a fixed-effect model using PLINK v1.07 (Purcell et al., 2007).

### Transcriptional Analysis of *EFCAB13*

Peripheral blood mononuclear cells (PBMC) were isolated by density gradient centrifugation using Lympholyte-H (Cedarline, Burlington, NC, United States) and stored at −80°C with RNAlater (QIAGEN, GmbH, Hilden, Germany) for RNA preservation. RNA was extracted using an RNeasy Plus Mini Kit provided by QIAGEN. The synthesis of cDNA was performed using a GoScript Reverse Transcription System (Promega). We performed two different PCR reactions:

1. Amplification specific for isoform B of *EFCAB13* (ENST00000517484): we performed at first RT-PCR by GoTaq® DNA polymerase (primers: forward: 5′-GAT​GAC​TTT​GTA​AAT​GCT​CTC​GC-3′, reverse: 5′-CCT​GTG​GTA​GAG​GAT​GGT​CCT-3′), followed by an hemi-nested PCR using this reverse: 5′-CGT​GTT​GCT​CAT​CAT​AGT​ATC​A-3’.
2. Amplification of both A and B *EFCAB13* transcripts: we performed a single-step RT-PCR by GoTaq® DNA polymerase (primers: forward: 5′-GAT​GAC​TTT​GTA​AAT​GCT​CTC​GC-3′, reverse: 5′-CCG​TGT​TGC​TCA​TCA​TAG​TAT​CA-3′). cDNA sequencing analysis was performed using BigDye Terminator Cycle Sequencing Kit version 3.1 (Applied Biosystems) on an automated 3730 DNA analyzer (Applied Biosystems, Foster City, CA, United States). In order to perform ddPCR, 50 ng of RNA was reverse-transcribed using an iScript cDNA Synthesis kit (Bio-Rad Laboratories, Inc, United States). The ddPCR mix was prepared as follows: 10 μL of 2x ddPCR Supermix for EvaGreen (Bio-Rad Laboratories, Inc, United States), 1 μL of each primer [2 µM]: *EFCAB13* forward: 5′-GCG​TTG​CCT​GGT​GTC​ATT-3’; *EFCAB13* reverse: 5′-TCA​CCA​GCT​TCA​GTC​AGT-3’; *HPRT1* forward: 5′-ACC​CTT​TCC​AAA​TCC​TCA​GC-3’; *HPRT1* reverse: 5′-GTT​ATG​GCG​ACC​CGC​AG-3’; 6 μL of DNAase-free water, and 2 μL of cDNA (5 ng in total). The 20 μL droplet digital PCR (ddPCR) reaction mixture was then loaded into a sample well of a DG8 Cartridge for QX200 Droplet Generator (Bio-Rad Laboratories, Inc., United States), followed by 70 μL of Droplet Generation oil for probes into the oil wells, according to the QX200 Droplet Generator instruction manual. The generated droplets were transferred into a clean 96-well plate. The plate was placed on a thermal cycler and amplified to the endpoint, following these thermal conditions (ramp 2°C/s): step 1: 95 °C 5′, step 2: 95 °C 30”, 58 °C 1′ for 40 cycles, step 3: 4 °C 5′, step 4: 90 °C 5′, step 5: 4 °C 40′, 4 °C hold.

After PCR, the 96-well PCR plate was loaded on a QX200 Droplet Reader (Bio-Rad Laboratories, Inc., United States), which reads the droplets from each well of the plate. The analysis of the ddPCR data was performed with QuantaSoft analysis software version 1.7.4, which accompanied the QX200 droplet reader.

### In Silico Characterization of *EFCAB13*

For the *EFCAB13* gene, no reports are currently retrievable from major functional annotation databases such as Gene Ontology, KEGG, and Reactome. We queried the COEXPEDIA repository (Yang et al., 2017) for genes co-expressed with *EFCAB13*: this database currently contains approximately 8M co-expressions inferred from 2,622 series of microarray Affymetrix-based transcriptomic experiments. The co-expression maps generated by COEXPEDIA have the distinctive feature of being constructed within each study and thus within a specific biomedical context; furthermore, it reports associations between co-expressions and Medical Subjects Headings (MeSH) terms which index the published PubMed article related to the transcriptomic experiment.

Moreover, we mined the GEO database (http://www.ncbi.nlm.nih.gov/geo) with the goal of evaluating the *EFCAB13* profile in studies contrasting transcriptional profiles in MS patients with controls (Nickles et al., 2013; Acquaviva et al., 2020; Gandhi et al., 2010; Irizar et al., 2012; Iparraguirre et al., 2020). Our criteria for study inclusion were as follows: 1) a minimum sample size of 20 in both groups, 2) MS patients should not be under treatment, and 3) samples should be collected from peripheral blood (whole blood or PBMC). For each of the included studies, we identified the Illumina^®^ or Affymetrix^®^ probe for the *EFCAB13* expression measurement, converted values on the log_2_ scale to improve normality, and performed an inverse-variance meta-analysis of SMD between cases and controls under a random-effect model using R package *meta* (Balduzzi et al., 2019). One GEO series (Iparraguirre et al., 2020) was left out of meta-analysis and separately analyzed since the expression levels were measured with the RNA-seq technology.

### Ethics Statement

The study was conducted according to the guidelines of the Declaration of Helsinki and approved by the Ethics Committee of “Maggiore della Carità” Hospital of Novara, protocol codes CE 149/20 (approved: June 12, 2020) and CE 38/05 (approved: July 1, 2005), and by the Ethics Committee of the Foundation “San Raffaele” (protocol codes: MSGENE02, SMPP01, SMGENE01, FINGO-MS, and BANCA-INSPE).

Informed consent was obtained from all subjects involved in the study.

## Results

A total of 4,732 subjects of the Italian continental origin (2,390 MS patients, 2,342 healthy controls, HC) were investigate for this study. Patients and controls derived from three independent cohorts: discovery, replication (both pool-sequenced), and an array-based cohort.

### Discovery

We performed targeted sequencing on 49 pools of MS patients (N = 588) and 34 pools of healthy controls (N = 408), whose demographic and clinical data are reported in Table 1.

**TABLE 1:** Features of the three cohorts.

		Discovery		Replication		Array cohort	
		MS	HC	MS	HC	MS	HC
N	—	588	408	504	504	1,298	1,430
Sex	% females	60.33	57.84	68.05	32.14	66.49	33.08
Disease course	% BOMS	90.10	—	90.59	—	79.79	—
	% PPMS	9.90	—	9.41	-	20.21	—
Age at onset (years)	Mean	29.97	—	30.31	—	32.19	—
	Min–max	10–65	—	10–62	—	10–69	—
	SD	10.03	—	9.96	—	10.80	—
Disease duration (years)	Mean	11.33	—	11.68	—	10.18	—
	Min–max	0–45	—	0–43	—	0–44	—
	SD	8.16	—	8.66	—	7.28	—
EDSS	Median	2.5	—	2	—	2	—
	Range	0–9.5	—	0–9.0	—	0–9.5	—

Clinical and demographic features of MS patients, and HC, for the three cohorts; BOMS, bout-onset MS, PPMS, primary progressive MS, EDSS, expanded disability status scale; SD, standard deviation.

The study was conducted hypothesizing that the contribution of rare variants to MS susceptibility could be harbored within loci tagged by common variants identified within international GWAS efforts in MS: we thus selected those regions identified as associated with MS in the international meta-analysis (International Multiple Sclerosis Genetics Consortium, 2013), also showing a nominal association in the Italian cohort. This strategy yielded 46 regions, harboring 98 genes (Supplementary Table S1).

We used a bioinformatic pipeline whose parameters were chosen and tested in the context of a previous study from our group (Anand et al., 2016). After duplicate removal, we obtained an average of 13.9 and 11.0 million reads mapped on the NCBI human reference genome (build GRCh37) for the MS and HC cohorts, respectively. The mean depth was 361x (range: 219x–814x) for the MS cohort and 338x (range: 262x–464x) for the HC cohort, with no significant difference (*t*-test, *p* = 0.229) among cohorts, with 98% of the targeted regions covered by NGS reads in each pool. We report in Supplementary Figure S1 A–B bar plots depicting mean depth and coverage for MS and HC pools in the discovery cohort.

A total of 27,529 SNVs were called, of which we retained for downstream analyses only those passing filters and having a phred-scale QUAL score >100 (N = 16,203). Of these, 13,291 had MAF<5% and 12,178 had MAF<1%: among the 13,291 rare- and low-frequency variants, 32.5% were annotated as intergenic, 44.3% were intronic, 1.2% were mapped on ncRNA, 7.8% were exonic, 9 and 0.92% were in 3′ UTR and 5’ UTR regions respectively, and about 3% were located within 1,000 bases upstream of the transcription start site or downstream of the transcription end site, according to ANNOVAR annotation.

We applied a set of seven filters (see Methods) to evaluate the distinct contribution of synonymous, missense, nonsense, and regulatory SNVs. The overall gene-wise association statistics for four of the seven filters involving rare variants are shown in quantile–quantile plots depicted in Supplementary Figure S2.

The list of all the variants observed in the discovery cohort and passing the seven filters is reported in Supplementary Table S2, together with the functional annotation and the *in silico* predictors and regulatory annotations used to classify the variants within each of the filters.

We tested the gene-based contribution of variants to risk by means of the WSS burden test (Madsen and Browning, 2009) and the variance component C-alpha test (Neale et al., 2011), which is more flexibly sensitive to the bidirectional effect of SNVs. We next combined the two approaches by means of a “hybrid” test (Greco et al., 2016).

The different numbers of SNVs yielded by each filter are reported in Table 2, which also indicate the number of genes carrying at least two filtered SNVs on which the mutational burden of SNVs with two gene-wise tests was evaluated. Table 2 shows that *EFCAB13* (EF-hand calcium binding domain 13), a gene located on chromosome 17, was the gene with the highest level of association for all but the *Reg_01* filter, for which *VMP1* and *NPEPPS* were the top-associated genes according to the C-alpha and WSS tests, respectively.

**TABLE 2:** Number of SNVs and the top-associated gene in each of the seven filters in the discovery cohort.

			WSS			C-alpha		
Filter	No. of SNVs	Tested genes	Top gene	No. of SNVs	P	Top gene	No. of SNVs	P
*Disr*	17	1	*EFCAB13*	8	<0.0001	*EFCAB13*	8	0.0023
*MisDisr_01*	598	75	*EFCAB13*	13	0.001	*EFCAB13*	13	0.021
*MisDisr_05*	624	77	*EFCAB13*	16	<0.0001	*EFCAB13*	16	0.007
*DamDisr_01*	367	58	*EFCAB13*	7	0.0093	*EFCAB13*	7	0.0125
*DamDisr_05*	381	61	*EFCAB13*	10	0.0002	*EFCAB13*	10	0.0054
*SynMisDisr_01*	598	83	*EFCAB13*	22	0.0012	*EFCAB13*	22	0.022
*Reg_01*	1,393	84	*NPEPPS*	21	0.0213	*VMP1*	141	0.0011

We then combined the burden and variance component tests with a hybrid test. We adopted an exploratory approach and nominated for follow up in the replication phase those genes detected as nominally significant in any of the seven scenarios according to the hybrid test. We found 17 such genes, which are reported in Table 3: the genes that exhibited the highest level of association were again the *EFCAB13* gene (*p* < 0.0001 in *Disr*, *MisDisr_0*5, and *DamDisr_05* filters) and *SKAP2* gene (*p* < 0.0001 in *DamDisr_05* and *DamDisr_01* filters). As regarding rare variants with putative regulatory annotation, *NPEPPS* was the most significant gene (*p* = 0.0006).

**TABLE 3:** Genes nominated for replication.

Symbol	Chr	Disr	MisDisr_01	MisDisr_05	DamDisr_01	DamDisr_05	SynMisDisr_01	Reg_01
*TGFBR3*	1	—	**0.0266**	**0.0266**	0.6824	0.6824	0.0723	0.8001
*RPAP2*	1	—	0.3668	0.3668	—	—	**0.0495**	**0.0211**
*FBXO40*	3	—	0.7969	0.7969	0.5683	0.5683	0.5977	**0.0086**
*ETV7*	6	—	0.5763	0.5100	0.8311	0.8311	**0.0417**	0.2600
*SKAP2*	6	—	**0.0230**	**0.023**	**<0.0001**	**<0.0001**	**0.0230**	0.8813
*TAGAP*	6	—	0.0727	0.3667	0.1780	0.1780	**0.0498**	0.9128
*ELMO1*	7	—	0.1757	0.1757	0.1757	0.1757	**0.0199**	0.6864
*ZC2HC1A*	8	—	0.5695	0.5695	0.7601	0.7601	0.5426	**0.0495**
*MYC*	8	—	0.2675	**0.0356**	0.7708	**0.0066**	0.1548	0.5452
*STAT5A*	11	—	0.9656	0.9656	0.6077	0.6077	**0.0071**	0.5679
*CLECL1*	12	—	0.5840	**0.0461**	—	—	0.584	0.5223
*TM9SF2*	13	—	1	1	—	—	0.1918	**0.0050**
*SOCS1*	16	—	—	—	—	—	—	**0.0281**
*EFCAB13*	17	**<0.0001**	**0.0001**	**<0.0001**	**0.0011**	**<0.0001**	**0.0001**	0.8669
*NPEPPS*	17	—	0.6933	0.6933	0.6933	0.6933	0.2196	**0.0006**
*TUBD1*	17	—	**0.0415**	**0.0415**	0.3283	0.3283	0.2611	0.6951
*SCO2*	22	—	0.9688	0.9688	0.9688	0.9688	**0.0048**	—

*p*-values for the six filters for the genes that were nominated for replication. *p*-values were computed upon the application of the hybrid test in the discovery cohort. For nomenclature of filters, see the Methods section. Significant associations at *p* < 0.05 for the various filters are labeled in bold.

The complete set of association statistics for 98 genes in seven filtering scenarios is reported in Supplementary Table S3.

### Replication

The 17 genes identified in the discovery phase were sequenced in a second independent cohort of 504 pool-sequenced MS and 504 pool-sequenced HC using the same analytic pipeline. For the six genes that showed a significant burden with the regulative variants filter *Reg_01* (*RPAP2*, *FBX O 40*, *ZC2HC1A*, *TM9SF2*, *SOCS1*, and *NPEPPS*), we sequenced coding and UTR regions and intronic regions annotated as regulative, whereas for the others, we sequenced only the coding regions. Supplementary Figure S1 C–D shows bar plots depicting the mean depth and coverage for MS and HC pools in the replication cohort.

**FIGURE 1:**
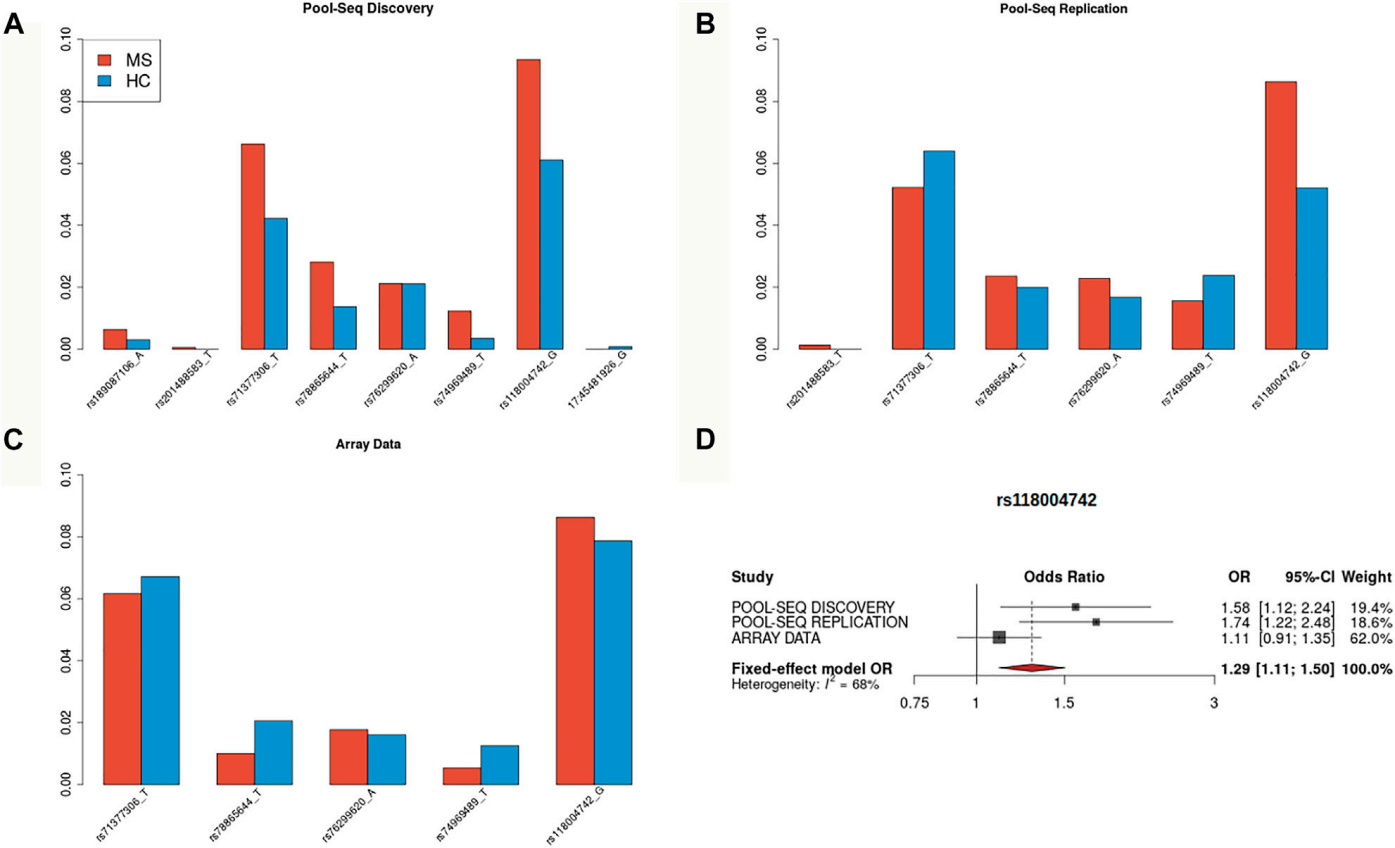
Bar plots of minor allele frequency for *EFCAB13* disruptive variants. Bar plots reporting minor allele frequencies for SNVs classified as disruptive (stop-loss, stop-gain, and splice site) located in the *EFCAB13* gene for the three investigated cohorts. Red: MS patients, blue: healthy controls. **(A)** discovery cohort (N = 8 SNVs), **(B)** replication cohort (N = 6 SNVs), **(C)** array-based cohort (N = 5 SNVs), and **(D)** forest plot for meta-analyzed odd ratios estimated for stop-gain SNV rs118004742.

After duplicate removal, we obtained an average of 4.4 and 4.3 million reads mapped on the NCBI human reference genome (build GRCh37) for the MS and HC cohorts, respectively. The mean depth was 543x (range: 173x–1,270x) for the MS cohort and 511x (range: 78x–1,572x) for the HC cohort, with more than 99% of the targeted regions covered by NGS reads in each pool. After applying the same quality control filters, we observed 636 variants, of which 554 had MAF<5% and 514 MAF<1%. Among the SNVs with MAF<5%, 27% were missense, 0.9% (N = 6) were non-sense, 15.4% were synonymous, and 21% were located in UTR regions. Out of 17 genes that were nominally associated in the hybrid test in the discovery cohort, two (*EFCAB13* and *MYC*) replicated at a nominal level on the replication cohort (*EFCAB13* for the *Disr* filter, *MYC* for the *MisDisr_05* filter). In particular, six disruptive variants in the *EFCAB13* gene were called among those already observed in the discovery cohort (Figures 1A,B): the overall impact of these variants was significantly associated with the MS status according to the hybrid test (*p* = 0.014), although the association was mostly driven by a bidirectional unbalance of allele frequencies as detected by the C-alpha test (*p* = 0.0062), rather than the burden test (*p* = 0.329). Conversely, the significant results of the hybrid test for the *MYC* gene (*p* = 0.0227) were mainly driven by a burden of missense variants (WSS test *p* = 0.0043) including only one variant with a similar trend in the discovery (Supplementary Figure S3), while the C-alpha test was not significant (*p* = 0.901).

### Follow Up of *EFCAB13* and *MYC* Variants on Array Data

The gene-level association for disruptive variants located in *EFCAB13* was tested in an additional independent cohort of Italian subjects, composed of 1298 MS and 1430 HC, genotyped on array platforms. Five out of the six SNVs jointly called in pool-seq discovery and replication cohorts, were available on array data (stop-gain SNVs rs71377306, rs78865644, rs74969489, rs118004742, and splice acceptor variant 76299620, see Figure 1C). These SNVs are low-frequency variants (MAF<0.05), with the exception of rs71377306 and rs118004742 (MAF = 0.058 and MAF = 0.065 in European non-Finnish, respectively). The MAFs for the variants are reported in Figures 1A–C.

Regarding *EFCAB13*, upon 10,000 permutations of the disease status, we did not find evidence of association in terms of burden of disruptive SNVs (WSS test, *p* = 0.199) but found an association for unbalance in the variance component approach (C-alpha test, *p* = 0.0219). The stop-gain SNV rs118004742 was the only variant showing a consistent pattern of association across the three cohorts since it was more frequent in the MS patients than in the HC (Figures 1A–C): on this SNV, an inverse-variance weighting meta-analysis between cohorts was performed under a fixed-effect model, yielding a summary OR = 1.29 at *p* = 10^–3^ (Figure 1D)**.** Intriguingly, the rs118004742 variant showed a nominal significant association (*p* = 0.04 OR = 1.012) in the international large-scale meta-analysis performed by IMSGC (International Multiple Sclerosis Genetics Consortium, 2018).

The same pipeline applied to the *MYC* gene did not allow us to test a gene-level association because only one of the SNVs jointly called in pool-seq discovery and replication cohorts was available on array data (rs4645959). However, a meta-analysis of effect sizes for this missense variant across the cohorts yielded no significant association (*p* = 0.072, OR = 1.22, under a fixed-effect model, Supplementary Figure S3).

Altogether, these results prompted us to perform further investigations only on the *EFCAB13* gene and in particular on the MS-associated stop-gain SNV rs118004742.

### In Silico Characterization of *EFCAB13*

No reports are currently retrievable for the *EFCAB13* gene from major functional annotation databases such as Gene Ontology, KEGG, and Reactome. To gain some insight on the putative function of the *EFCAB13* gene, we adopted a “guilt-by-association” method, a classical approach for poorly studied genes, by investigating which genes are co-expressed with the study gene. The ranking of the co-expressed genes from the COEXPEDIA resource (Yang et al., 2017) by the log-likelihood score (LSS, Supplementary Table S4) identified *NF1* (neurofibromin 1) as the gene with the highest co-expression. The most significantly enriched GO biological process was *Negative Regulation of Cell Migration* (*p* = 5.24*10^–4^), whereas as of MESH terms, we found that the top three terms enriched for *EFCAB13* coexpression were *Immune System Diseases*, *Th1 Cells,* and *Th17 Cells*, all reaching a score of 6.63.

To attempt a preliminary investigation of the possible role of *EFCAB13* in MS, we screened GEO repository for transcriptional assays involving MS and HC and identified six studies (date: December 22, 2020) according to our inclusion criteria (see Methods and Supplementary Table S5). One study (GSE159225) was conducted with RNA-seq and separately analyzed by means of the *t*-test, whereas for the five microarray studies (GSE41850 discovery, GSE41850 replication, GSE136411, GSE17048, and GSE41890) (Nickles et al., 2013; Acquaviva et al., 2020; Gandhi et al., 2010; Irizar et al., 2012; Iparraguirre et al., 2020), the standardized mean differences (SMDs) for the *EFCAB13* expression level between MS and HC were combined by means of a random-effect meta-analysis. Overall, data from 319 MS patients and 174 HC were evaluated: meta-analysis yielded an estimated 95% CI [–0.4132, 0.0993] for the effect size with *p* = 0.23 and heterogeneity I2 = 39.3%, thus revealing a non-significant down-regulation of the gene in MS patients. This trend in down-regulation was also observed in data on counts from RNA-seq study (*p* = 0.23, delta = -44).

### Transcriptional Analysis of *EFCAB13*

**FIGURE 2:**
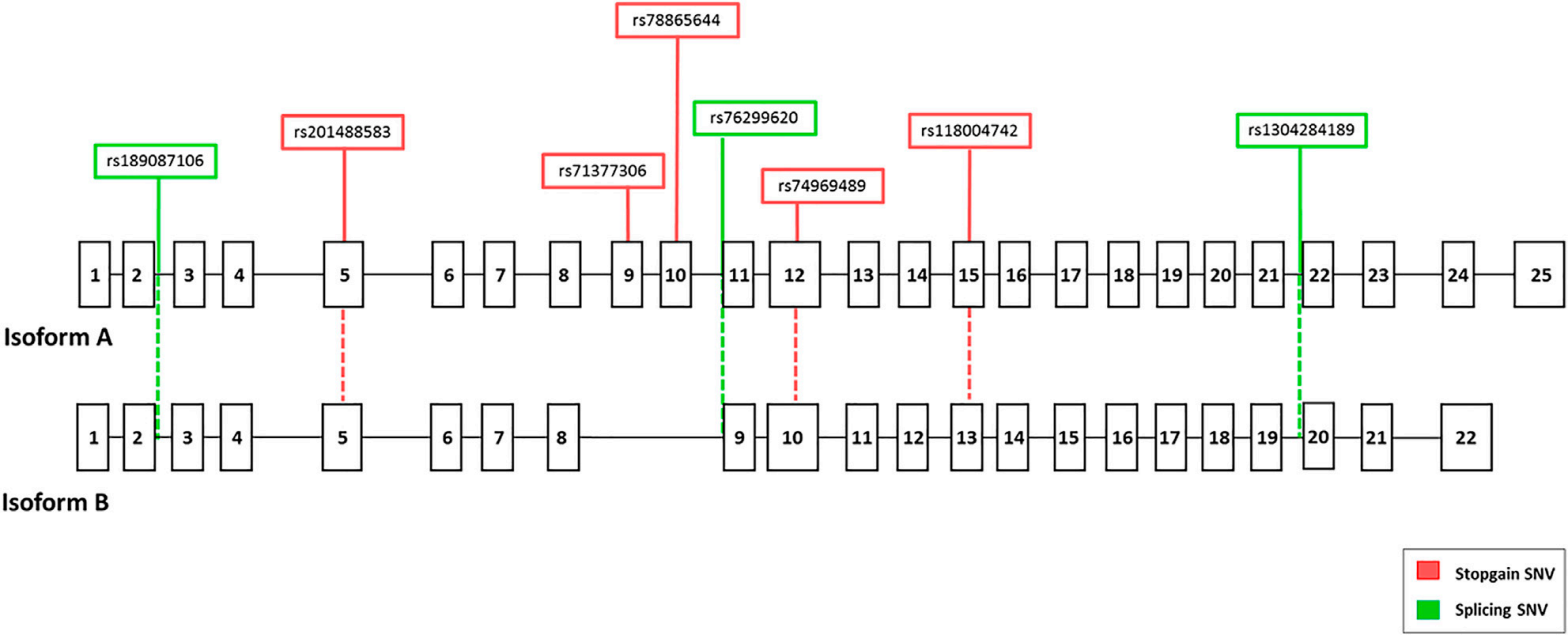
Genomic structure of the two main isoforms of the *EFCAB13* gene. GeneBank references (Ensembl) of *EFCAB13* isoforms are ENST00000331493 (Isoform A) and ENST00000517484 (isoform B), respectively. The positions of the eight disruptive variants are displayed in red (stop-gain variants) or green (splicing variants).

To investigate a possible effect of the associated stop-gain SNV rs118004742 variant on the *EFCAB13* transcript, we amplified and sequenced *EFCAB13* from the cDNA of 14 individuals (9 HC and 5 MS) heterozygous for this variant. Among the 13 transcripts reported on Ensembl and GTEx databases for the gene, we focused on the two transcripts that are actually translated into proteins according to the Uniprot database: isoform A, the longest isoform of 3,957 bp (ENST00000331493), and isoform B, of 3,466 bp (ENST00000517484) (Figure 2).

**FIGURE 3:**
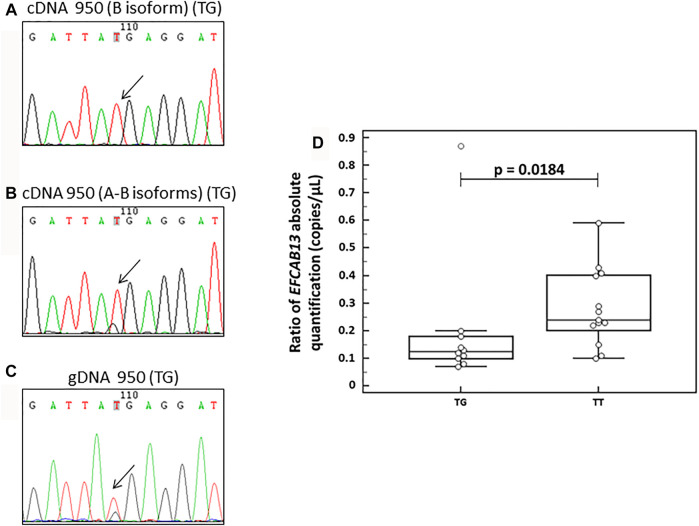
Analysis of the *EFCAB13* transcript**. (A–C)** Electropherograms derived from Sanger sequencing on a heterozygous healthy subject for the variant rs118004742. (**A)** Sequence derived from RT-PCR designed to amplify cDNA of only isoform B of the *EFCAB13* transcript; **(B)** sequence derived from RT-PCR designed to amplify cDNA of both A and B *EFCAB13* transcripts; **(C)** sequence derived from PCR on genomic DNA of the same subject. The rs118004742 variant is indicated by an arrow. **(D**) *EFCAB13* cDN*A* expression levels by ddPCR analysis. Box plots display the expression levels by ddPCR analysis in 23 subjects, including 10 carriers (TG) and 13 non-carriers (TT) of the stop-gain rs118004742-G variant. The ddPCR assay tests the expression of both A and B *EFCAB13* isoforms. The *p*-value was derived from the Mann–Whitney test.

Upon specific amplification of the B isoform, we observed only the presence of the common allele (T) in the cDNA (Figure 3A). This evidence can lead us to hypothesize a possible degradation of the transcript carrying the rare stop-gain allele by a non-sense-mediated decay mechanism, possibly leading to a down-regulation of *EFCAB13* at RNA. These data are also supported by the evidence that the carriers of the stop-gain variant have a lower expression of this gene by droplet digital PCR (ddPCR) analysis (10 carriers vs 13 non-carriers, *p* = 0.0184) (Figure 3D). Surprisingly, the presence of the rare allele was detected in the cDNA amplification that did not discriminate among the two isoforms (Figure 3B), similar to the amplification on the genomic DNA (Figure 3C).

## Discussion

The present study was focused on the investigation of the impact on MS risk of SNVs in the low-frequency portion of the allelic spectrum, with a pooled-sequencing approach that allowed a cost-effective sequencing of a target panel of 98 genes. This panel was constructed from loci identified in MS from large-scale international GWAS, thus postulating that genes within such common loci could be candidates enriched with rare variants contributing to MS risk.

Since the single-variant analysis is under-powered in typical sample sizes like ours, we adopted a gene-based strategy to evaluate the cumulative effect of SNVs under multiple genetic architectures.

We selected the most significantly associated genes according to a test which combines the results from both unidirectional and bidirectional unbalance of risk alleles, allowing for risk and protective effects, and sought to replicate their significance in an independent replication cohort. Our data showed that the strongest signal of association in the discovery cohort was observed for variants of one gene, *EFCAB13*, under multiple filters and consistently replicated at a gene level in two independent cohorts of pool-sequencing data and array data. In particular, we had suggestive evidence of association of disruptive variants in *EFCAB13*, which should be pursued further in larger cohorts. Among disruptive variants in *EFCAB13,* the stop-gain SNV rs118004742 showed the most consistent pattern of statistically significant association among the three cohorts and combining the entire cohort of 2390 MS patients and 2342 HC in the meta-analysis.

*EFCAB13* encodes for the EF-hand calcium binding domain 13 protein, which is a poorly characterized calcium binding adapter protein. It is broadly expressed in over 22 tissues including blood, although at minor levels compared to other tissues (Fagerberg et al., 2014). Our preliminary RT-PCR experiments showed that *EFCAB13* is expressed in PBMC, activated T cells, and dendritic cells (data not shown), and our query search on co-expressed genes revealed an interesting enrichment of MESH terms which refer to immunological themes including *Th1 Cells* and *Th17 Cells* terms, which could suggest a possible involvement in these processes.

The associated stop-gain variant rs118004742 falls into an EF-hand 2 domain (524–559 aa), a structural domain involved in calcium ion binding. To better elucidate the functional consequences of the associated variant on *EFCAB13* mRNA transcripts, we conducted RT-PCR and sequenced the cDNA of heterozygous individuals for this variant, focusing on the two transcripts that are actually translated into proteins according to the Uniprot database: isoform A (ENST00000331493) and isoform B (ENST00000517484). From this analysis, we did not find the presence of the isoform B transcript with the minor stop-gain allele. Therefore, we can speculate a possible degradation of this transcript by a non-sense-mediated decay mechanism led by the stop-gain allele, predicting a down-regulation of RNA and hence of the protein level. These data were also supported by the evidence that the carriers of the stop-gain variant had a lower expression of *EFCAB13* mRNA transcripts by ddPCR analysis. Since we detected the presence of minor stop-gain in the cDNA amplification that did not discriminate among the two isoforms, we can hypothesize an isoform-dependent influence of the stop variant in the transcript degradation. Intriguingly, we observed a trend towards a down-regulation of the *EFCAB13* transcript in MS in comparison with controls in six transcriptional assays from public GEO repository; it is thus tempting to speculate that a down-modulation of *EFCAB13,* partially associated with its genetic variants, may be involved in MS susceptibility. Since the function of this gene is largely unknown, further studies are needed to understand the possible role of a reduced expression of *EFCAB13* in MS pathogenesis.

The strength of our study resides in the fact that analyses were carried out on a relatively homogeneous Italian population, which is a desirable setting in rare variant association studies, and we performed a thorough investigation of various genetic architectures through application of various filtering scenarios. To better define the role of rare variants in MS susceptibility, we chose to apply several filtering criteria using different frequency thresholds and various functional classes. This strategy allowed us to separately analyze the contribution of variants with a putative high impact on the protein function from that of variants with a putative milder impact. Furthermore, the fact that 17 genes have been fully sequenced (including intronic and intergenic portions) allowed us to design a filter that took into account the role of rare variants with a putative regulative effect as well based on annotations that specifically predict the possible influence of a variant on gene expression. Although the first decade of GWAS provided overwhelming evidence that genetic signals for complex traits reside within the non-coding portion of the genome, likely influencing traits by perturbing gene regulation and through rewiring of regulatory networks (Maurano et al., 2012; Farh et al., 2015), rare variants in non-coding regions are still poorly studied in gene-based studies.

A possible limitation of this study resides in the fact that coding variants have been classified on the basis of their predicted impact on the protein function, not considering the functional domains of the protein. Thus, there is a chance that the contribution of domain-specific variants has been un-noticed.

Even though the power is higher than that of the corresponding single-variant tests and this strategy has been previously successfully employed in studies of size comparable to ours (Ionita-Laza et al., 2012; Shtir et al., 2016; Kumar et al., 2020), the power for gene-based tests for sample size comparable with ours is modest (<50% at *α* = 10^–4^) across a range of genetic architectures as evidenced by a comprehensive simulation study (Moutsianas et al., 2015).

Despite power limitations, our study is to our knowledge the largest one focusing on the contribution of rare and low-frequency SNVs to MS risk in a homogeneous population with an NGS-based identification of variants in a large number of MS genes. Overall, we observed a modest contribution of rare variants to risk in the target established MS loci, a result that is consistent with what was reported by a previous effort in large cohorts (Hunt et al., 2013) on the low contribution to heritability of rare variants in known susceptibility loci for six autoimmune diseases, including a lower number of MS loci. Indeed, it should be mentioned that the recent large-scale meta-analysis performed by IMSGC (International Multiple Sclerosis Genetics Consortium, 2018) revealed a far more limited number of associated rare coding variants as compared to the corresponding IMSGC genome-wide study (International Multiple Sclerosis Genetics Consortium, 2019) and a lower amount of disease heritability explained by rare variants.

This is in contrast with examples of other complex diseases for which rare or low-frequency variants seem to play a larger role, as demonstrated for the low-frequency variants in *PCSK9* associated with a high effect size with low LDL and protection against coronary heart disease (Cohen et al., 2006), for variants contributing to type 2 diabetes heritability together with common variants within the same genes (Mahajan et al., 2018) and variants which segregate non-randomly with a common variant, creating a “synthetic association” captured by a common variant within GWAS array (Nicolas et al., 2018).

These papers and our effort also underline the importance of the increasing availability of large reference panels like the Haplotype Reference Consortium (McCarthy et al., 2016) and population-specific panels (Huang et al., 2015) that will allow reliable imputation of low-frequency variants to assess their contribution to risk in complex diseases.

In conclusion, although the largest studies focused on rare-low frequency support the concept that low-frequency variants play a less relevant role in comparison with common variants in MS and explain a lower amount of disease heritability, we propose the role of disruptive low-frequency variants in a poorly characterized gene whose replication and further functional studies may reveal novel disease mechanisms.

## Members of the PROGEMUS (PROgnostic GEnetic factors in MUltiple Sclerosis) Group

**P. Crociani** [Deprtment of Medical Sciences, Neurology Unit, IRCCS Casa Sollievo Della Sofferenza, San Giovanni Rotondo (FG), Italy]; **D. Vecchio** (MS Center, Neurology Unit, Maggiore della Carità Hospital, Novara, Italy); **P. Ragonese** (MS Center, Neurology Unit, Policlinico Paolo Giaccone, Palermo, Italy); **A. Gajofatto** (MS Center, Neurology Unit, Policlinico Borgo Roma, Verona, Italy); **E. Scarpini** (MS Center, Neurodegenerative and Demyelinating Diseases Unit, Foundation IRCCS Ca’ Granda Ospedale Maggiore Policlinico, Milan, Italy); **A. Bertolotto**, [MS Center, Neurology Unit, S. Luigi Gonzaga Hospital, Orbassano (TO), Italy]; **D. Caputo** (MS Center, IRCCS Fondazione Don Carlo Gnocchi, Milan, Italy); **C. Gasperini** (MS Center, Department of Neurosciences, S. Camillo Forlanini Hospital, Rome, Italy); **F. Granella** (Department of General and Specialized Medicine, Neurology Unit, Parma Hospital, Parma, Italy); **S. Cordera** (MS Center, Neurology Unit, Parini Regional Hospital, Aosta, Italy); **P. Cavalla** (MS Center, Department of Neurosciences, S. Giovanni Battista–Molinette Hospital, Turin, Italy); **R. Cavallo** (MS Clinic, S.G. Bosco Hospital, Turin, Italy); **R. Bergamaschi** (MS Interdepartmental Research Center, Department of General Neurology, Foundation C. Mondino National Neurologic Institute, Pavia, Italy); **G. Ristori** (MS Laboratory, Neurology Unit, S. Andrea Hospital, Rome, Italy); **C. Solaro** [Mons. L. Novarese Functional Recovery and Rehabilitation Center, Moncrivello (VC), Italy]; **F. Martinelli** [Neurology Unit, IRCSS S. Donato Policlinico, S. Donato Milanese (MI), Italy]; **F. Passantino** (MS Center, Department of Neurology, SS. Antonio and Biagio Hospital, Alessandria, Italy); **M. Pugliatti** (Department of Biomedical and Specialized Chirurgical Sciences, Neurology Clinic, University of Ferrara, Ferrara, Italy); **A. Gallo** (MS Center, I Neurology Clinic, II University of Neaples, Neaples, Italy); **L. Brambilla** (MS Centre, Neurology 4 Unit, Foundation IRCCS C. Besta Neurologic Institute, Milan, Italy); **M. Clerico** [MS Center, Department of Specialized and Nerologic Diseases, S. Luigi Gonzaga Hospital, Orbassano (TO), Italy]; **F. Capone** (Neurology Unit, Campus Biomedico Policlinico, Rome, Italy).

## Members of the PROGRESSO (Italian Network of Primary Progressive Multiple Sclerosis) Group

**F. Esposito**, **G. Liberatore**, **M. Rodegher**, **P. Rossi**, **M. Radaelli**, **L. Moiola** and **B. Colombo** (Department of Neurology, San Raffaele Hospital, Milan, Italy); **A. Ghezzi** and **P. Annovazzi** (Department of Neurology, S. Antonio Abate Hospital, Gallarate, Italy), **R. Capra** (Department of Neurology, Spedali Civili, Brescia, Italy), **G. Coniglio** (Department of Neurology, Madonna delle Grazie Hospital, Matera, Italy), **M. P. Amato** and **B. Nacmias** (Department of Neurology, University of Florence, Florence, Italy); **G. Tedeschi** and **A. D’Ambrosio** (Department of Neurological Sciences, Second University of Naples, Naples, Italy), **P. Cavalla** (Department of Neurology, University of Turin, Turin, Italy); **F. Patti** and **E. D’Amico** (Department DANA, G.F. Ingrassia, Neurosciences Section, Multiple Sclerosis Center, PO “G. Rodolico”, Catania, Italy); **D. Galimberti** and **E. Scarpini** (Department of Neurological Sciences, Centro Dino Ferrari, University of Milan, Fondazione Ca’ Granda, Ospedale Maggiore Policlinico, Milan, Italy); **P. Gallo** and **M. Atzori** (The Multiple Sclerosis Centre of Veneto Region, First Neurology Clinic, Department of Neurosciences, University Hospital of Padova, Padua, Italy); **L. Grimaldi** and **S. Bucello** (Division of Neurology, Fondazione Istituto San Raffaele “G. Giglio”, Cefalù, Italy); **G. Mancardi** and **E. Capello** (Department of Neuroscience, Ophthalmology and Genetics, University of Genova, Genoa, Italy).

## Data Availability

The original contributions presented in this study are included in the Supplementary Material and can be found in online repositories. The names of the repository/repositories and accession number(s) can be found below: https://www.ebi.ac.uk/eva/, PRJEB32114. Further inquiries can be directed to the corresponding authors.
