# Supplementary material for: Contribution of Rare and Low-Frequency Variants to Multiple Sclerosis Susceptibility in the Italian Continental Population
Source: Front Genet. 2022 Jan 3;12:800262. doi: 10.3389/fgene.2021.800262 (PMC8762330; doi:10.3389/fgene.2021.800262)

Figure S1 – Barplot showing mean depth and mean coverage of targeted regions across pools in discovery cohort (upper panels) and pools in replication cohort (lower panels).

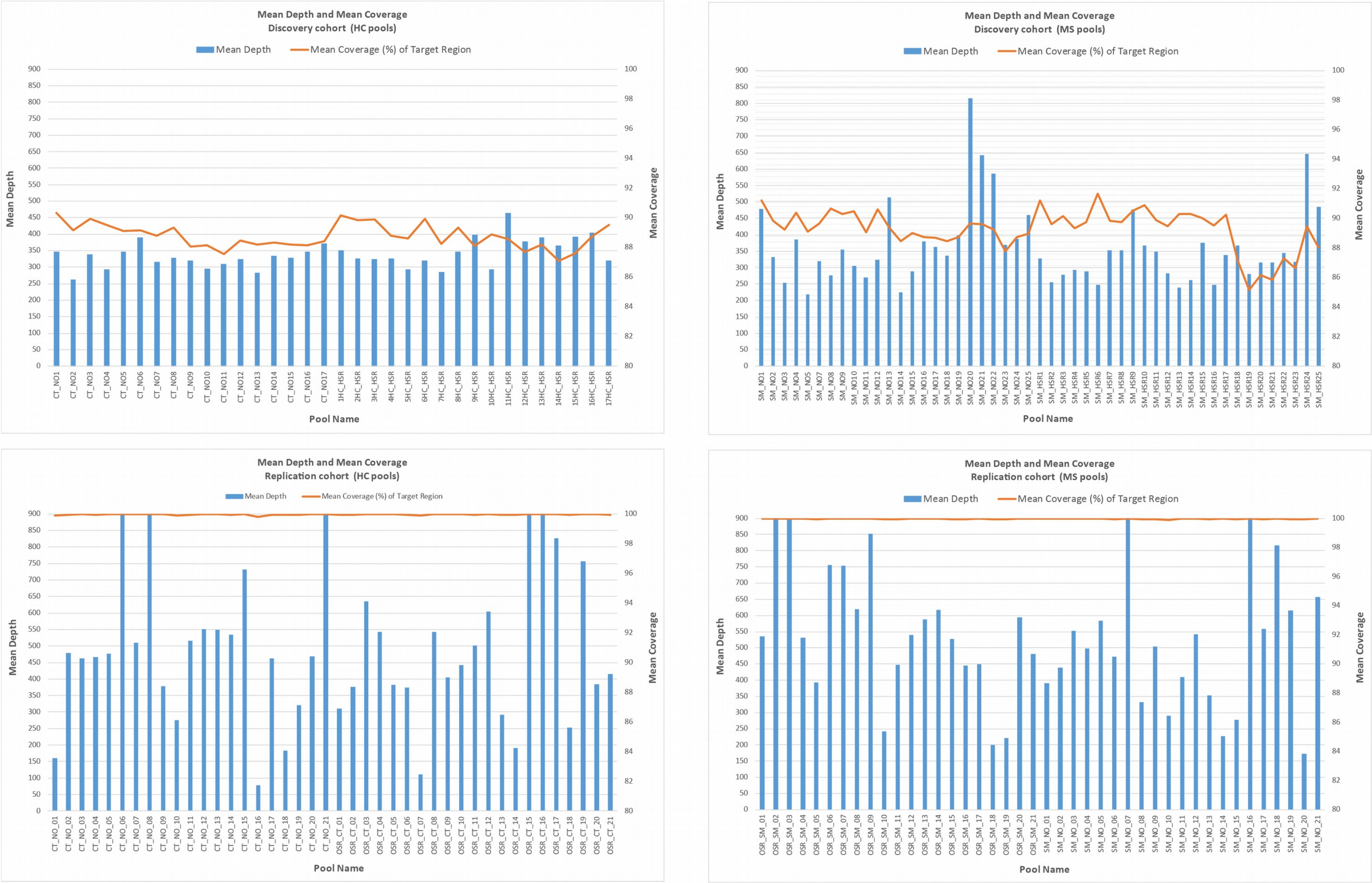

Figure S2 – Quantile-quantile plot of gene-based tests.

The plots depict deviations from the null (uniform distribution of p-values, represented by diagonal line) for the 98 candidate genes for the four selected filters. The plots are depicted both for burden (WSS) and variance component (C-alpha) tests.

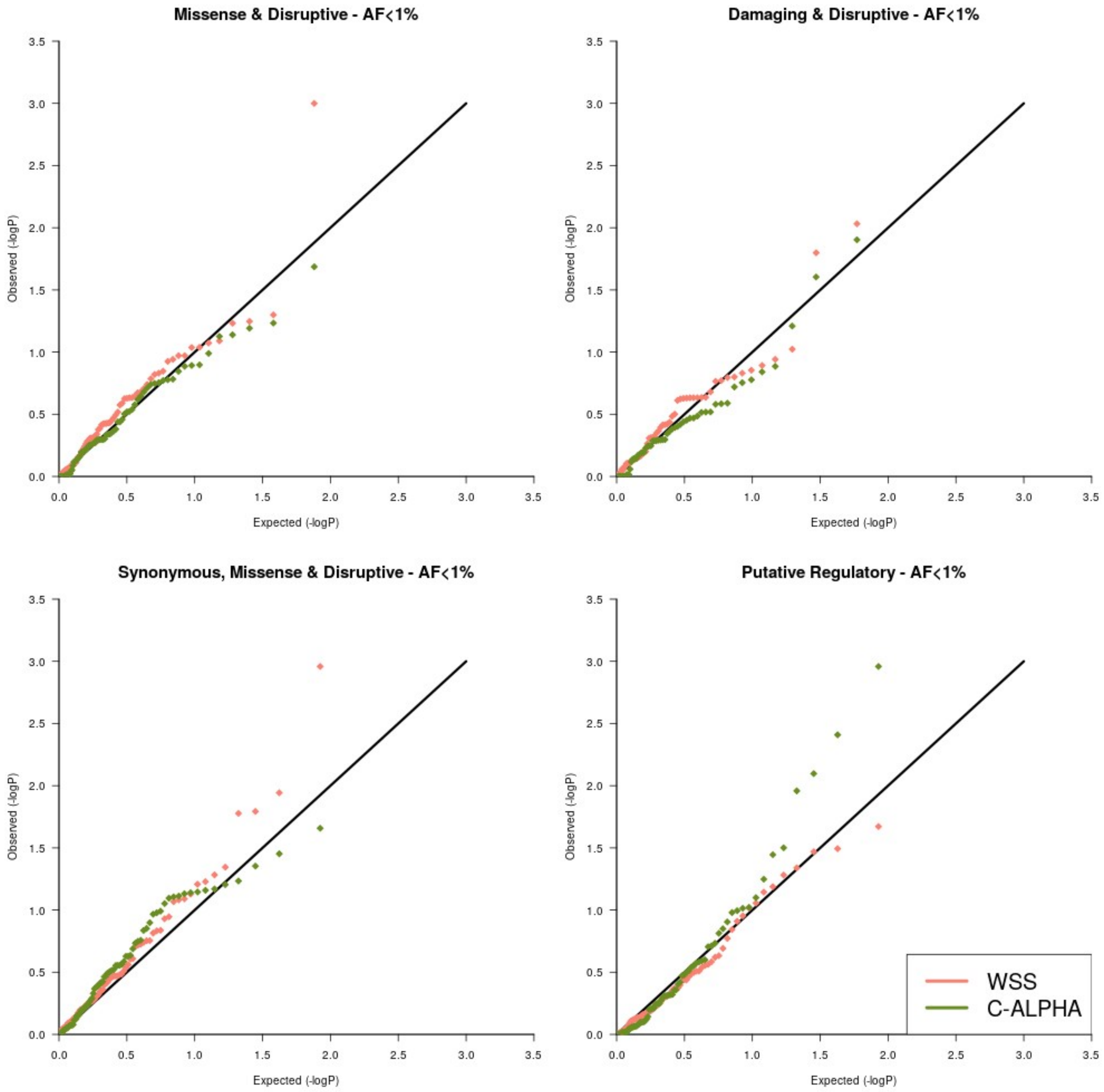

Figure S3 – Barplots of Minor Allele Frequency for *MYC* in *MisDisr_05* filter

Barplots reporting minor allele frequencies for SNVs classified as missense or disruptive (stop-loss, stop-gain, splice site) at MAF<5%, located in *MYC* gene, for the three investigated cohorts. Red: MS patients, blue: healthy controls. **a)** discovery cohort (N=6 SNVs); **b)** replication cohort (N=12 SNVs, of which 3 shared with discovery cohort); **c)** array-based cohort (N=1 SNV); **d)** forest plot of meta-analyzed odds ratios estimated for missense SNV rs4645959.

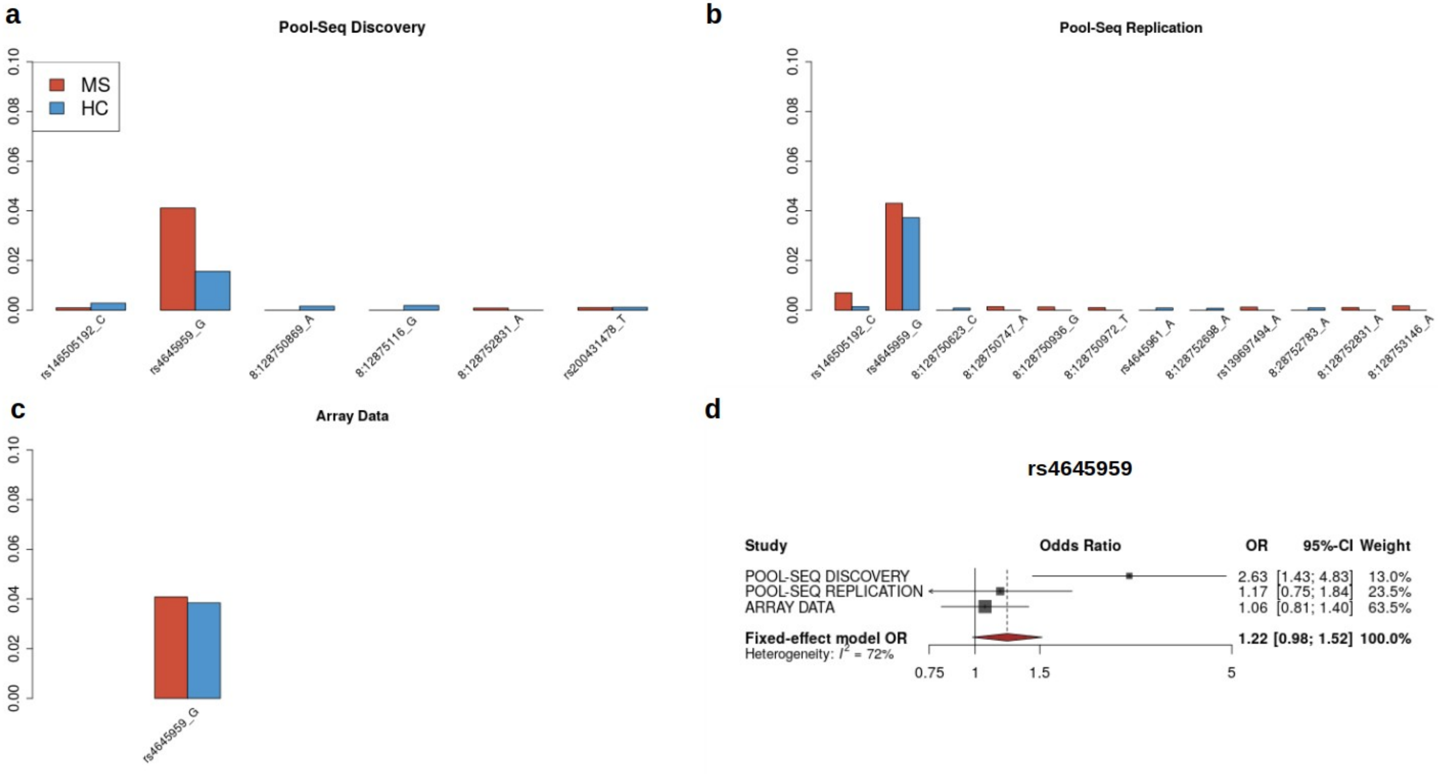

Supplement: Supplementary file 4 [file DataSheet1.PDF]
