# Supplementary material for: Contribution of Rare and Low-Frequency Variants to Multiple Sclerosis Susceptibility in the Italian Continental Population
Source: Front Genet. 2022 Jan 3;12:800262. doi: 10.3389/fgene.2021.800262 (PMC8762330; doi:10.3389/fgene.2021.800262)
Supplement: Supplementary file 1 [file DataSheet2.PDF]

UNIVERSITÀ DEL PIEMONTE ORIENTALE

Novara, 22th October 2021

Prof. Emmanuelle Genin

Editor in Chief

Frontiers in Genetics

Dear Prof. Emmanuelle Genin,

please find enclosed our manuscript entitled "*Contribution of rare and low-frequency variants to multiple sclerosis susceptibility in the Italian continental population*" that we submit for publication to Frontiers in Genetics as Original Article.

Recent genome-wide association studies identified over 200 risk loci for Multiple Sclerosis (MS) focusing on common variants, which account for about 50% of heritability. This study aimed to investigate whether low-frequency/rare functional variants may account for MS missing heritability by studying a total of 4,744 subjects of Italian continental origin (2402 patients with MS, 2342 healthy controls, HC) including a discovery cohort and two replication independent cohorts. To this purpose, we tested the cumulative effect (burden) of these variants by sequencing a large number of known MS loci (n=98) in 588 Italian patients with MS and 408 matched healthy controls (HC). Genes showing a significant burden were sequenced in an additional independent cohort of 504 MS and 504 HC. The strongest MS association signal in both cohorts was observed for a burden of loss of function variants (stop-gain, stop-loss or splicing variants) mapping in *EFCAB13*, a gene coding for a protein of unknown function containing calcium binding domains. The variant showing the largest difference in the two cohorts (rs118004742) was confirmed in a third independent cohort (1298 MS and 1430 HC, p=0.001) and tested for functional impact on *EFCAB13* expression. This is a stop-gain variant associated with a lower expression of *EFCAB13* gene, as a consequence of a transcript degradation by non-sense mediated decay.

Altogether, these results lead us to conclude that we identified a novel low frequency functional variant associated with MS susceptibility.

This study contains many elements of novelty shedding new light on the complex genetic architecture of MS:

- this is the first study which systematically searches for variants in MS patients and matched controls in a large number of MS loci, using a Next Geneneration Sequencing approach;
- this is the first study that identifies a novel low frequency functional variant associated with MS susceptibility with a possible functional role;

- the unknown function of the identified gene may pave the way to the identification of new pathogenetic pathways in MS.

The research presented in this manuscript is original and has not been published elsewhere nor is under consideration by other Journals.

None of the authors have competing financial interests related to this manuscript and all the authors fulfilled the criteria for authorship. This study was approved by local ethics committees, and written informed consent was obtained by all participants in the study. All relevant data can be found within the manuscript. Specific data can be available upon reasonable request.

We would like to thank you in advance for your consideration of our study.

Sincerely yours,

Sandra D'Alfonso and Filippo Martinelli Boneschi on behalf of the authors

Sandra D'Alfonso
Department of Health Sciences, Center on Autoimmune and Allergic Diseases (CAAD), UPO, University of Eastern Piedmont, A. Avogadro University, Novara, Italy, Via Solaroli 17, 28100 Novara; Italy. E-mail: sandra.dalfonso@med.uniupo.it

Filippo Martinelli Boneschi
Department of Pathophysiology and Transplantation (DEPT), Dino Ferrari Centre, Neuroscience Section, University of Milan &
Neurology Unit and MS Centre, Foundation IRCCS Ca' Granda Ospedale Maggiore Policlinico, Via Francesco Sforza 35, 20122 Milan, Italy. E-mail: filippo.martinelli@unimi.it
